# Subacute small bowel obstruction in a twin pregnancy: a diagnostic and management challenge

**DOI:** 10.1093/jscr/rjag281

**Published:** 2026-05-11

**Authors:** Katherine Gar Yen Lau, Christina K H Yu, Charlotte Frise

**Affiliations:** St. Mary's Hospital, Praed Street, Imperial College Healthcare NHS Trust, London W2 1NY, United Kingdom; St. Mary's Hospital, Praed Street, Imperial College Healthcare NHS Trust, London W2 1NY, United Kingdom; St. Mary's Hospital, Praed Street, Imperial College Healthcare NHS Trust, London W2 1NY, United Kingdom; Queen Charlotte's and Chelsea Hospital, Du Cane Rd, Imperial College Healthcare NHS Trust, London W12 0HS, United Kingdom

**Keywords:** small bowel obstruction, pregnancy, diagnosis, management

## Abstract

Small bowel obstruction (SBO) in pregnancy is rare but serious, presenting a diagnostic challenge due to symptom overlap with common gestational conditions and hesitancy around radiologic imaging. We describe a case of progressive, subacute SBO in a 46-year-old woman with dichorionic diamniotic twins and a history of caesarean section. The case culminated in bowel perforation, emergency laparotomy, and preterm delivery. This case highlights the need for early recognition, appropriate imaging, and multidisciplinary management to minimize maternal and foetal morbidity.

## Introduction

Small bowel obstruction (SBO) accounts for 15% of surgical admissions for acute abdominal pain in non-pregnant adults but is rare in pregnancy [1, 2]. Symptoms are often nonspecific, and concerns of foetal safety may delay imaging and management. Yet, delayed diagnosis significantly increases the risk of maternal morbidity and foetal loss. This case highlights the diagnostic challenges of SBO in pregnancy and the importance of early intervention.

## History

A 46-year-old woman with a dichorionic diamniotic twin pregnancy booked for antenatal care at 18 weeks’ gestation. Obstetric history included a previous caesarean delivery at 38 weeks for placenta praevia, complicated by 2.2 L haemorrhage and postnatal transient hypertension. Past medical history included asthma.

In this pregnancy, hypertension developed at 20 weeks requiring treatment with Methyldopa. Anomaly scan demonstrated two structurally normal foetuses with high placentas.

At 25 weeks’ gestation, she presented with vomiting and epigastric pain following a restaurant meal. With a presumed diagnosis of gastroenteritis, she was discharged following intravenous fluids and antiemetics. She re-presented with episodes of vomiting, epigastric pain, and diarrhoea and was again conservatively managed. Bilious vomiting prompted further investigations. Blood and urinalysis results are presented in Table 1. Abdominal ultrasound showed normal hepatic architecture, thin-walled gallbladder with sludge without calculi.

**Table 1:** Laboratory and urinalysis results at 25 weeks’ gestation. Values out of range are indicated (^*^).

Blood results	
Haemoglobin	123 g/L
Platelets	332 × 10^9^/L
White cell count	6.2 × 10^9^/L
Alanine transaminase	747^*^ IU/L
Aspartate transaminase	372^*^ IU/L
Bilirubin	24^*^ μmol/L
Albumin	29 g/L
C-reactive protein	35.7 mg/L^*^
Lactate dehydrogenase	310 U/L^*^
Lactate	0.6 mmol/L
Placental growth factor	441 pg/mL
Immunoglobulins	Normal
Autoantibody screen	Negative
Hepatitis A, B, C/Cytomegalovirus/Epstein–Barr Virus	Negative
Fibrinogen	6.55^*^ g/L
Prothrombin time	13.9 s
Activated partial thromboplastin time	32.7 s
**Urinalysis**	
Urine dipstick	4+ ketones, 1+ protein
Urinary protein:creatinine ratio	28 mg/mmol

Over the subsequent month, her symptoms resolved, and liver function improved. At 31 weeks’ gestation however epigastric pain and vomiting returned, with normal bowel opening. She reported preterm labour symptoms, with a shortened cervix on speculum examination. Maternal tachycardia was noted with otherwise normal observations. Foetal monitoring was initially pathological but subsequently normalized. Laboratory results showed: alanine transaminase 365 IU/L, creatinine 192 μmol/L, haemoglobin 126 g/L, white cell count 7.2 × 10^9^/L, C-reactive protein 160 mg/L with metabolic acidosis on an arterial blood gas. Intravenous rehydration was given, alongside magnesium sulphate and corticosteroids for foetal neuroprotection and lung maturity. Abdominal magnetic resonance imaging (MRI) was performed as the chronology meant the initial diagnosis of gastroenteritis was now unlikely. The MRI revealed a mechanical SBO involving the proximal to mid-ileum with a distinct transition point and no signs of perforation.

Nasogastric decompression and fluid resuscitation were initiated as first-line therapy. Due to the evolving clinical picture with foetal cardiotocography concerns, a balanced decision was made for delivery the following morning. Whilst awaiting her caesarean, the patient became suddenly unwell, with acute signs of peritonism and hypotension. Twin 1 developed a bradycardia and an emergency caesarean was performed.

A midline laparotomy was performed and a fresh small bowel perforation identified. A segment of mid-ileum was densely adherent to the anterior uterine and abdominal wall. Adhesiolysis was performed for access to the lower uterine segment. Following delivery of both twins, a 1 cm perforation proximal to a transition point was identified with oedematous bowel. A 20 cm bowel resection with side-to-side primary anastomosis was performed.

Postnatally, the patient required intensive care with vasopressors, nasogastric decompression, and total parenteral nutrition. Sepsis secondary to *Enterococcus faecalis* bacteraemia occurred. A large intra-abdominal collection and pleural effusion developed, both requiring drainage. A distal iliac vein thrombus was diagnosed incidentally necessitating 3 months of anticoagulation. The twins recovered well in the neonatal unit.

## Discussion

SBO in pregnancy is rare, affecting 0.001%–0.003% of pregnancies [2]. Adhesions from previous surgery are the leading cause, causing 70% of cases. Although the incidence of post-caesarean section SBO is low (0.1%–1.35%), rising rates of caesarean births may increase future incidence [3].

Diagnosing SBO during pregnancy is challenging, as symptoms are often non-specific and may mimic normal pregnancy. Classic features such as complete constipation are observed in only 6.1% of cases and typically abdominal pain (54.7%) and vomiting (39.2%) are the sole presenting symptoms [4]. In our case, significant adhesions were present following only one prior caesarean. The compressive effect of a twin gestation may have further increased the risk of obstruction, with the intermittent nature of subacute SBO likely contributing to a delayed diagnosis.

In this case, raised liver enzymes were the presenting biochemical abnormality. Two prior case reports have described isolated raised liver transaminases, where bowel obstruction is thought to have prevented the duodenal bile passage [5, 6]. The cause here remained unclear and resolved postnatally.

Outside of pregnancy, abdominal radiographs (AXR), and computed tomography (CT) scans are primary used for SBO diagnosis. Water-soluble contrast has been used safely in pregnancy with an additional therapeutic effect in adhesional SBO but requires AXRs for imaging [4, 7]. Guidelines recommend foetal radiation exposure should remain below 50 mGy [8, 9] due to both deterministic effects on foetal cellular development as well as stochastic injury to cellular genetic material. Deterministic effects only occur above 100 mGy, but stochastic effects are seen with exposures over 25 mGy, with increased rates of childhood cancers above baseline reaching significant levels above 50 mGy. A simple AXR gives negligible foetal exposure of 1.0 mGy of radiation, whilst an abdominal CT yields radiation levels of 25 mGy [10], well within the radiation guidance. Non-ionizing imaging is preferred in pregnancy to negate all radiation risk, but may be more costly and not readily available, with lower detection rates. Ultrasound detects up to 55% of cases [11], MRI 67% [12], and CT 94% [13]. Imaging modality choice should therefore reflect acuity of clinical presentation, resource availability, and patient wishes.

SBO without foetal compromise does not necessarily warrant immediate delivery. However, those managing women with SBO should be aware of the higher risk of foetal complications such as growth restriction, preterm delivery, antepartum haemorrhage. Close monitoring of these pregnancies should continue with a multi-disciplinary approach [14].

Management should follow standard principles: fluid resuscitation, electrolyte correction, nasogastric decompression, and early surgical input. Complications of SBO in pregnancy are significant, including preterm delivery (45%), maternal mortality (3.7%), and foetal mortality (17%) [4, 15]. Delaying surgery increases these risks. Therefore, in pregnancy, earlier surgical intervention should be considered than in non-pregnant populations. Nutritional support is essential to minimize complications such as foetal growth restriction and maternal metabolic complications.

## Conclusion

SBO should be suspected even if symptoms are non-specific, particularly when the history is not consistent with an alternative diagnosis. Timely imaging and early intervention are essential to reducing maternal-foetal adverse outcomes.
